# Supplementary material for: Embodied social interaction constitutes social cognition in pairs of humans: A minimalist virtual reality experiment
Source: Sci Rep. 2014 Jan 14;4:3672. doi: 10.1038/srep03672 (PMC3890942; doi:10.1038/srep03672)
Supplement: Supplementary Information [file srep03672-s1.doc]

Embodied social interaction constitutes social cognition in pairs of humans: A minimalist virtual reality experiment

Tom Froese, Hiroyuki Iizuka, and Takashi Ikegami

**Supplementary information**

There are some additional methodological issues, which may have had an influence on the results of our experiment and therefore must be made explicit.

1) Participants’ free-text feedback about the design of the study revealed that the true nature of the non-responsive moving object was often an explicit source of concern and confusion. Some players believed that they had to avoid interacting with a computer-controlled artificial agent, while others wondered whether it was a pre-recorded movement that could be copying their own behaviour from a previous trial. During post-experiment debriefing it appeared that no player had suspected that there was a rigid link between the other’s avatar and the moving object. In hindsight it would have been better to clarify the precise nature of the shadow object in advance, because players’ uncertainty about the capacities of the moving object made many overly cautious about whether they were in fact interacting with their partner. This could have reduced the probability to click on the avatar.

2) The need for an explicit instruction to cooperate was derived from insights gained during pilot studies, which showed that cooperation was not the default style of interaction. Some players would instead assume they were playing a competitive game, and would therefore try to detect the other player without being detected. And even without any explicit intention to compete, some players would just loose their motivation for genuinely continuing with an interaction after they had already clicked, thereby making it impossible for the other player to succeed if they had not clicked already. We therefore decided to increase pro-social motivation and to deflect the unwanted tendency for players to try to compete with each other into a competition between teams by running the study as a team-based tournament competition.

Nevertheless, it could still be occasionally observed that some players behaved differently just before or directly after clicking, for example by pausing extensively (see, e.g., the blue player in Fig. 3), and with some even reaching for the post-trial questionnaire before remembering that the trial was still on-going and then once again starting to use the trackball. This kind of interruption must have had a detrimental effect on the other player, who might have just been about to click as well, but was then inexplicably faced with a non-responsive object. This could have reduced the likelihood of Joint Success, and may have increased players’ uncertainty in subsequent trials as well. In other words, although the clicks are useful because they are an explicit marker of a subjective state, an implicit marker would eliminate this disruption by a factor that is external to the interaction process.

3) Some participants complained that occasionally they were just about to click when a trial finished (see, e.g., player 2’s trial 13 and perhaps also trial 15 in Table 2). Indeed, participants only received feedback about the remaining time once, namely 30 seconds after the start of a trial. Continuous feedback about the time remaining in a trial might have led to less absent clicks. Some participants requested longer trial durations, but this was not found to be necessary. In fact, a related study found significant results with only 30 second trials^1^.

4) In the original perceptual crossing paradigm the shadow’s distance to the avatar was specified as 48 units of space, but an agent-based model of this setup raised the worry that a fast-moving player could recognize that two quickly successive contact stimuli usually indicate the presence of the other and its shadow^2^. We therefore followed the suggestion of that modelling study to place the shadow at a distance of 150 units, which is the furthest possible distance from the avatar (placing the shadow object at the other side of the environment, at a distance of 300 units, would have the undesired effect of creating another stable situation of perceptual crossing, whereby players could interact with each other via their shadow objects).

However, this slight modification had the effect of highlighting an unexpected drawback of the perceptual crossing paradigm when compared to the related “double video TV monitor” paradigm proposed by Trevarthen and colleagues^3^. To single out social contingency, the latter setup introduces a playback of past behaviour that was recorded during a live interaction, whereas the former setup introduces a playback of on-going behaviour that, when it is actually encountered, cannot be the same kind of behaviour that would have been performed by the other participant during a live interaction (for the simple reason that the other’s avatar and shadow cannot be at the same location at the same time: thus, if a player makes contact with the other’s shadow this necessarily means that the shadow is copying the behaviour that the other player performs while not in mutual contact). Results of a study based on the double video paradigm indicate that the detection of a responsive presence is more complicated when using playback behavior^1^. Nevertheless, even though the different styles of movement exhibited by the other’s avatar and its shadow may contribute to the accuracy of clicks in the perceptual crossing paradigm, crucially players were still unable to consciously appropriate this difference in previous studies.

1. Iizuka, H., Ando, H. & Maeda, T. The anticipation of human behavior using "parasitic humanoid". in *Human-Computer Interaction: Ambient, Ubiquitous and Intelligent Interaction* (ed J. A. Jacko) 284-293 (Springer-Verlag, 2009).

2. Froese, T. & Di Paolo, E. A. Toward minimally social behavior: Social psychology meets evolutionary robotics. in *Advances in Artificial Life: Darwin Meets von Neumann. 10th European Conference, ECAL 2009* (eds G. Kampis, I. Karsai, & E. Szathmáry) 426-433 (Springer-Verlag, 2011).

3. Murray, L. & Trevarthen, C. Emotional regulations of interactions between two-month-olds and their mothers. in *Social Perception in Infants* (eds T.M. Field & N.A. Fox) 177-197 (Ablex Publishing, 1985).
